# A multicentre interventional study to assess blood-borne viral infections in Belgian prisons

**DOI:** 10.1186/s12879-021-06405-z

**Published:** 2021-07-27

**Authors:** Dana Busschots, Cécile Kremer, Rob Bielen, Özgür M. Koc, Leen Heyens, Christian Brixko, Pierre Laukens, Hans Orlent, Pascal Bilaey, Francis De Smet, Geert Hellemans, Gaetan Muyldermans, Luk Van Baelen, Niel Hens, Hans Van Vlierberghe, Geert Robaeys

**Affiliations:** 1 Faculty of Medicine and Life Sciences, Hasselt University, Hasselt, Belgium; 2 Department of Gastroenterology and Hepatology, Ziekenhuis Oost-Limburg, Genk, Belgium; 3 Interuniversity Institute for Biostatistics and statistical Bioinformatics (I-Biostat), Data Science Institute, Hasselt University, Diepenbeek, Belgium; 4 School of NUTRIM, Maastricht University Medical Centre, Maastricht, the Netherlands; 5 Department of Gastroenterology and Hepatology, CHR Citadelle, Liège, Belgium; 6 Dienst Coördinatie Medische Zorg, Federale Overheidsdienst Justitie, Brussel, Belgium; 7 Department of Gastroenterology and Hepatology, AZ St-Jan, Bruges, Belgium; 8 Department of Gastroenterology and Hepatology, UZ Gent, Ghent, Belgium; 9 Epidemiology and Public Health, Sciensano, Brussels, Belgium; 10 Centre for Health Economic Research and Modelling Infectious Diseases, Vaccine and Infectious Disease Institute, University of Antwerp, Antwerp, Belgium; 11 Department of Gastroenterology and Hepatology, University Hospitals KU Leuven, Leuven, Belgium

**Keywords:** Prison, Hepatitis C, Hepatitis B, HIV, Blood borne viral infections, Screening, Recommendations

## Abstract

**Background:**

Prevalence data on viral hepatitis B (HBV), hepatitis C (HCV), and HIV infection in prison are often scarce or outdated. There is currently no systematic screening for these blood-borne viral infections (BBV) in Belgian prisons. There is an urgency to assess the prevalence of these BBV to inform policymakers and public healthcare.

**Methods:**

This was a multicentre, interventional study to assess the prevalence of BBV using opt-in screening in prisons across Belgium, April 2019 – March 2020. Prisoners were tested using a finger prick and BBV risk factors were assessed using a questionnaire. A generalized linear mixed model was used to investigate the association between the various risk factors and HCV.

**Results:**

In total, 886 prisoners from 11 Belgian prisons were screened. Study uptake ranged from 16.9 to 35.4% in long-term facilities. The prevalence of HCV antibodies (Ab), hepatitis B surface antigen (Ag) and HIV Ab/Ag was 5.0% (44/886), 0.8% (7/886), and 0.2% (2/886). The adjusted odds for HCV Ab were highest in prisoners who ever injected (*p < 0.001*; AOR 24.6 CI 95% (5.5–215.2). The prevalence of detectable HCV RNA in the total cohort was 2.1% (19/886). Thirteen (68.4%) prisoners were redirected for follow-up of their HCV infection.

**Conclusions:**

Opt-in testing for viral hepatitis B, C and HIV was relatively well-accepted in prisons. Compared with the general population, prisoners have a higher prevalence of infection with BBV, especially for HCV. Systematic screening for these BBV should be recommended in all prisons, preferably using opt-out to optimize screening uptake.

**Trial registration:**

Retrospectively registered at clinical trials NCT04366492 April 29, 2020.

**Supplementary Information:**

The online version contains supplementary material available at 10.1186/s12879-021-06405-z.

## Background

At any given moment, an estimated 1.6 million individuals are imprisoned throughout the European member states [1]. In Belgium, approximately 10.800 people were incarcerated in 2020 [2].

Chronic hepatitis C virus (HCV) infection is a global health problem urging the World Health Organization (WHO) to set elimination goals by 2030 by reducing new hepatitis B virus (HBV) and HCV viral infections by 90% (specifically 80% reduction in new HCV cases) and mortality by 65%. One of the key populations for HCV infection are prisoners, where the prevalence is substantially higher than in the general population [3–6]. The HCV antibody (Ab) and RNA prevalence in the general population is, respectively, 1.0 and 0.3% in Belgium [7]. In Europe, the prevalence of HCV Ab in the general population ranges from 0.5% in Western Europe to 6% in Eastern Europe [8]. In prisons, the estimated prevalence is 15.5% in Western Europe and 20.2% in Eastern Europe [5]. These high rates of HCV infection in prisoners and the substantial risks associated (e.g., cirrhosis) with untreated HCV infection emphasizes the importance of HCV screening and access to treatment in prisons [9]. Therefore, the WHO advises testing all prisoners for HCV infection [10].

However, compliance with WHO guidelines for HCV screening in prisons remains insufficient worldwide. In Western Europe, only ten (34%) of the 29 surveyed countries reported HCV screening programs for prisoners in 2010 [9, 11]. Since then, only scarce data have been published on the extent to which evidence-based HCV recommendations have been implemented in prisons, either in Europe or globally [12, 13]. In the United Kingdom, opt-out screening in prisons was introduced as early as 2014 [14]. Implementing this procedure is crucial, as it has been shown that opt-out screening increases the uptake of screening in prisons [15–17]. Furthermore, various studies have shown that improved screening with opt-out procedures and subsequent treatment with direct-acting antivirals (DAAs) is cost-effective [18–21]. Therefore, widespread HCV screening procedures and treatment of incarcerated populations (treatment-as-prevention) would contribute to achieving the goal of global HCV elimination by 2030 [21–23].

Prisoners also have a disproportionately high burden of HBV viral infection [5, 24]. In a systematic review by the European Centre for Disease Prevention and Control (ECDC), the prevalence of hepatitis B surface antigen (HBsAg) in prison ranged from 0.3 to 25.2%. In contrast, the prevalence of HBsAg in the general population ranged from 0.1 to 4.4% [8]. In Belgium, one study reported an overall HBsAg prevalence of 1.0% at an emergency department of a large educational hospital in 2017 [25]. Beyond this, there is a scarcity of data on HBV viral infection in prisons, not only in Belgium but worldwide [4].

In Belgium, 0.3% of the general population has been diagnosed with HIV between 1982 and 2016 [26]. Data on HIV prevalence in Belgian prisons is scarce. Still, the prevalence in European prisons is estimated at 5.0% (95% CI 0.0–11%), indicating that incarcerated people also have a disproportionately high burden of HIV [5, 27]. Incarceration can disrupt HIV care for people who were being treated in the community [28]. Antiretroviral therapy can be interrupted by arrest and detention, depending on its availability [29]. This can be prolonged due to stigmatization and discrimination against HIV-positive prisoners, causing a denial of their HIV-positive status [30, 31].

In Belgium, healthcare among the general population depends on the different Ministers of Healthcare, each acting either at the federal or state levels. However the legal authority overseeing healthcare in prisons lies with the Minister of Justice at the federal level. Nevertheless, healthcare is free for all prisoners in Belgium. In addition, every prisoner should have access to the necessary care, medication, and follow-up as described in the Nelson Mandela Rules of the United Nations [32].

Prevalence data on HCV, HBV, and HIV in prison is scarce and outdated in Belgium. Furthermore, there is currently no systematic screening program for these blood-borne viral infections (BBV). Therefore, measuring the prevalence could give us a better impression of the current challenges concerning these BBV in prison. In addition, these data are necessary to inform policymakers and public healthcare, not only in Belgium but also in other countries/regions with similar epidemiological and jurisdictional healthcare systems.

## Materials and methods

### Study settings

There is currently no systematic screening for HCV, HBV, or HIV in all prisons in Belgium. Only three out of 35 prisons have an extensive medical service with the presence of a specialist. Screening for BBV could be carried out at the local prison. However, for the follow-up of an infection, transfer to another prison is mandatory. Concerning a chronic HCV infection, direct-acting antiviral treatment can only be prescribed and initiated by a hepatologist. In addition, harm reduction programs are present in Belgian prisons however these do not include needle syringe programs.

### Study design

This study was a multicentre, interventional cohort study assessing the prevalence of HCV, HBV, and HIV using opt-in screening in prisons between April 2019 and March 2020 in Belgium. Eleven out of 35 prisons throughout Belgium were pre-selected by the Federal Public Service Justice to participate in the study. In all prisons, there were both national and foreign prisoners. However, in only two of these prisons, there was a women’s section. Security status varied both between prisons and within prisons ranging from an open regime to a closed and high-security regime. Screening was performed in five pre-trial departments and eight long-term detention departments. Participants were included if they were aged 18 years or older and provided written informed consent. Participants were excluded if they could not provide written informed consent (language barrier, illiteracy) or if they had already participated in the study (re-entry within the inclusion period in one of the prisons). All participating prisoners could withdraw from the investigation at any time after admission, without any consequences for further treatment or their sentence.

### Data collection

In the pre-trial departments, screening was performed by the prison medical staff. The screening was only carried out on weekdays, 5 days a week. Every first person who entered the prison system was asked to participate. If the prisoner refused to participate or was unable to participate, the next person who entered the system was asked. When a prisoner refused to participate, the reason for the refusal was asked and noted. The prisoners were tested immediately after informed consent was given. Although the medical staff is part of the prison system, it was made clear to each participant that the answers provided on the questionnaire were for the study only and would not be used for any other purpose. Additionally, the medical record is strictly separated from the legal record. In order to minimize the workload of the medical staff, only one person per day was included.

A study team from Hasselt University (UH) carried out screening in the long-term detention departments. The UH study team conducted the study independently of the prison authorities and was not part of the prison staff. The study team visited the prisons on predefined dates. Two weeks before the study visit, prisoners were informed of the study by posters and could register through the prison medical staff. On the day of the study visit, the study team went over the consent form with each participant where upon consent, the prisoner was immediately tested.

#### Screening

All screenings were performed in the same manner. Prisoners were tested for HCV Ab, HBsAg, and HIV by a finger prick test. For HCV Ab detection, the OraQuick® was used, for HBsAg the HBsAg Rapid device®, and Alere™ HIV combo® to test for HIV Ab/Ag. While waiting for the rapid test results, BBV risk factors were assessed through a face-to-face questionnaire. The questionnaire was available in Dutch, French, English, Arabic, Spanish, Italian, Romanian, and Russian and covered 20 questions. Data from the questionnaire included birth gender, year of birth, country of birth (if not Belgium subdivided in low to moderate (0–1.3%) and moderate to high (1.3–6.7%) viremia for HCV [33]), questions related to incarceration, ever having lived together with an infected person, sexual preference, number of unsafe sexual partners, tattoos or piercings placed, age of first drug use, kind of drugs used and when (ever, past 6 months), frequency of drug use (during the past 6 months), ever injecting drug use (IDU), kind of IDU and when (ever, past 6 months), frequency of IDU (during the past 6 months), having shared injecting paraphernalia and receiving opioid agonist therapy (OAT) in prison.

#### Follow-up

If the participant tested negative for HCV, HBV, and HIV, no further follow-up was required. The participant was then informed about the risks and primary prevention methods available in prison. In case of a positive test, an additional short questionnaire was made available to the prison staff. The questionnaire’s data included referral to the prison physician and whether the prisoner showed up at the appointment. In addition, we also requested if an ongoing viral infection was detected after blood sampling. In fact, during the initial study screening, finger prick testing was only performed for HCV Ab, HBsAg and HIV Ab/Ag. Data on the follow-up of a positively tested prisoner were collected retrospectively 1 month after screening.

#### Patient consent statement

The study was approved by the Ethical Committees of UZ Gent (2018/0780) and Hasselt University. The study protocol is registered at clinicaltrials.gov (NCT04366492). The study was conducted in accordance with the provisions of the Declaration of Helsinki and its amendments. Good clinical practice guidelines were followed throughout the study and all participants provided written informed consent.

### Endpoints of the study

This study’s primary endpoint was to measure the prevalence of BBV (HCV/HBV/HIV) in Belgian prisons using an opt-in screening method. The secondary objective was to analyse the risk factors associated with these BBV and analyse the uptake of counselling by a prison physician in Belgian prisons.

### Statistical analyses

Patient demographics were summarized using mean ± interquartile range for continuous characteristics and by proportions for categorical characteristics.

To account for heterogeneity between individuals from the different prison sites (pre-trial vs. long-term detention), a generalized linear mixed model (GLMM) was used to investigate the association between the various risk factors and HCV. In these models, the prison site was then included as a random intercept. Univariate models were used to assess the association for each risk factor separately. Risk factors associated (*p* < .150) to HCV Ab in these univariate analyses were included as fixed effects in a multiple GLMM. The model reduction was done in a backward stepwise manner based on the .05 significance level. Due to the low numbers of HBV, HCV RNA and HIV positivity, risk factor analyses were only performed for HCV Ab. All analyses were performed using R version 3.6.3.

#### Sample size

The ideal sample size for a prevalence study is a function of the expected prevalence and precision for a given confidence level [34]. For a small prevalence, as is the case for HCV Ab, a conservative choice for the amount of precision has to be made using one-fifth of the estimated prevalence (for the effect size) [35].

In this study, an estimated HCV Ab prevalence of 10% was used. For a confidence level of 95%, z is 1.96. P is 0.10 and d = 0.10/5 = 0.02 (the formula is provided in [see Additional file 1]), where z is the quantile of the normal distribution corresponding to the level of confidence, P is the expected prevalence, and d is the effect size (i.e., the maximum difference between estimated and true prevalence). Therefore, the inclusion of 864 prisoners was necessary. However, since the prevalence will be estimated in a study using data from different prisons (cluster design), the design factor was considered [36]. Therefore, the sample size was multiplied by a factor of 1.5 [36]. Therefore, a total of at least 1.296 prisoners had to be included.

## Results

Between April 2019 and March 2020, 886 prisoners from 11 Belgium prison sites were screened for HCV Ab, HBsAg, and HIV using a finger prick. Study uptake ranged from 16.9 to 35.4% in long-term facilities. In pre-trial detention, 80/309 (25.9%) of the prisoners who were asked to participate refused to participate due to various reasons such as being scared of the finger prick (10/80, 12.5%), not interested in participating (35/80, 43.8%), language barrier (16/80, 20.0%), already tested outside (16/80, 20.0%), or worried about privacy (3/80, 3.8%). The prisoners who refused were not included in any further analyses. The socio-demographic characteristics of the study population are shown in Table 1.

**Table 1:** Socio-demographic characteristics of the study population and the results of the univariate and multiple generalized linear mixed models analyses, *n* = 886

			Univariate		Multivariate	
Characteristics		n (%)	***P***-value	OR (95% CI)	***P***-value	AOR (95%CI)
**Birth year** (mean ± IQR)		1981 ± 1974–1990	0.161	1.0 (1.0–1.0)		
**Gender**	Male	825 (93.1)		ref		
	Female	61 (6.9)	0.487	0.6 (0.1–2.1)		
**Site of inclusion**	Pre-trial detention	229 (33.7)	0.552	1.3 (0.5–3.8)		
	Long term detention	587 (66.3)		ref		
**Country of birth**	Belgium	612 (69.1)		ref		
	Other	271 (30.6)	**0.022**	**0.4 (0.1–0.8)**		
	*Low-moderate HCV prevalence*	*224 (82.7)*				
	*Moderate-high HCV prevalence*	*47 (17.3)*				
	*Missing*	*3 (0.3)*				
**Incarcerated before**	Yes	566 (63.9)	**0.004**	**3.4 (1.6–8.4)**		
	No	316 (35.7)		ref		
	*Missing*	*4 (0.5)*	*–*	*–*		
**Ever lived together with a person having HCV/HBV/HIV**	Yes	52 (5.9)	**< 0.001**	**11.7 (5.5–24.4)**	**0.011**	5.6 (1.5–22.4)
	No	834 (94.1)		ref		ref
**Sexual preference**	Heterosexual	826 (93.2)		ref		
	Homosexual	18 (2.0)	0.971	1.0 (0.1–5.5)		
	Bisexual	26 (2.9)	0.472	1.7 (0.3–6.4)		
	*Missing*	*16 (1.8)*	*–*	*–*		
**Number of partners with whom there have ever been unsafe sexual contacts** (*n* = 611)	1–4	265 (43.4)	ref	ref		ref
	5–9	129 (21.1)	0.563	1.4 (0.4–4.4)	0.440	2.0 (0.3–12.4)
	> 10	217 (35.5)	**0.017**	**3.0 (1.3–7.5)**	**0.031**	4.6 (1.3–21.8)
**Tattoo**	None	450 (50.8)		ref		
	Safe	237 (26.7)	**0.038**	**2.3 (1.0–5.3)**		
	Potentially non-sterile	199 (22.5)	**0.003**	**4.1 (1.9–9.0)**		
**Age first drug use non-IDU** (mean ± IQR)		17.5 ± 14–18	0.556	1.0 (0.9–1.0)		
**Used drugs last 6 m** (*n* = 633)	Yes	435 (68.7)	0.113	1.9 (0.9–4.3)		
	No	198 (31.3)		ref		
**Frequency use** (*n* = 435)	Less than once a week	132 (30.3)		ref		
	More than once a week	169 (38.9)	0.555	0.8 (0.3–1.9)		
	Daily	133 (30.6)	0.991	1.0 (0.4–2.4)		
	*Missing*	*1 (0.2)*				
**Used heroin last 6 months**	Yes	44 (10.6)	**< 0.001**	**11.0 (4.9–26.4)**	**< 0.001**	9.3 (2.6–37.6)
	No	370 (89.4)		ref		ref
**Ever IDU** (*n* = 633)	Yes	129 (20.4)	**< 0.001**	**38.0 (15.9–107.2)**	**< 0.001**	24.6 (5.5–215.2)
	No	503 (79.5)		ref		ref
	*Missing*	*1 (0.1)*	*–*	*–*		
**Ever shared materials for IDU** (*n* = 129)	Yes	54 (41.9)	**< 0.001**	**56.3 (22.5–170.1)**		
	No	75 (58.1)		ref		
**Frequency IDU last 6 m** (*n* = 129)	Never	98 (76.0)		ref		
	Less than once a week	9 (7.0)	**0.001**	**13.4 (2.5–62.2)**		
	More than once a week	10 (7.8)	**0.024**	**6.9 (1.0–34.0)**		
	Daily	12 (9.2)	**< 0.001**	**53.8 (14.1–251.2)**		

*Note*: ‘unsafe’ refers to sexual contact without the use of a condom

*Abbreviations*: *OR* odds ratio, *IQR* interquartile range, *HCV* hepatitis C virus, *HBV* hepatitis B virus, *IDU* injecting drug use

### Prevalence of blood-borne viral infections in Belgian prisoners

**Fig. 1:**
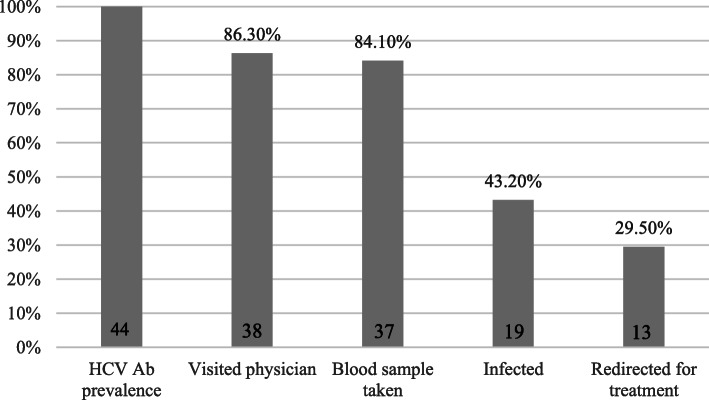
Cascade of care of hepatitis C in prison. Abbreviations:* HCV* hepatitis C virus, *Ab* antibody

Fifty-three (6.0%) prisoners tested positive on one of the three rapid tests (Fig. 1). There were no coinfections reported.

Forty-four (5.0%) of the prisoners tested positive for HCV Ab (Fig. 1). One prisoner who was referred for follow-up after a positive HCV Ab test refused to have a blood sample taken by the prison doctor. The prevalence of detectable HCV RNA in the total cohort was 19/886 (2.1%) (Table 2). Of these 19 prisoners, nine (47.4%) were unaware of their infectious status. Thirteen (68.4%) prisoners were redirected for HCV viral infection follow-up and the potential start of treatment. For four other prisoners, the length of stay in prison would be too short to start treatment, and two prisoners refused referral.

**Table 2:** Socio-demographic characteristics of the study population with a hepatitis C viral infection (*n* = 19)

Characteristics		n (%)
**Birth year** (mean ± IQR)		1980 ± 1973–1986
**Gender (male)**		19 (100%)
**Country of birth (Belgium)**		15 (78.9%)
**Ever lived together with a person having HCV/HBV/HIV (yes)**		5 (26.3%)
**Number of partners with whom there have ever been unsafe sexual contacts (*n*** **= 13)**	1–4	3 (15.8%)
	5–9	3 (15.8%)
	> 10	7 (36.8%)
**Heroin last 6 months (yes)**		8 (42.1%)
**Ever IDU (yes)**		18 (94.7%)
**OAT in prison (yes)**		10 (52.6%)

*Note*: ‘unsafe’ refers to sexual contact without the use of a condom

*Abbreviations*: *IQR* interquartile range, *HCV* hepatitis C virus, *HBV* hepatitis B virus, *IDU* injecting drug use, *OAT* opioid agonist therapy

Seven prisoners (0.8%) tested positive for HBsAg, of whom five were not aware of their status. One prisoner did not show up at the prison doctor for further follow-up. As a result, six out of seven prisoners were redirected to a specialist for follow-up of their HBV viral infection.

The two (0.2%) prisoners who responded tested HIV positive were not referred further after confirmation by the medical staff of ongoing antiretroviral treatment in both cases.

### Risk factors associated with HCV ab positivity

Most of the risk factors associated with HCV Ab positivity were related to IDU (Table 1). Harm reduction was present in Belgian prisons, with 83 prisoners receiving OAT during this study. The unadjusted odds for HCV Ab were highest in those who had ever shared materials for IDU (*p* < .001, OR 56.3 CI 95% 22.5–170.1). Prisoners born abroad were significantly less likely to be HCV Ab positive than those born in Belgium (*p* = .022, OR 0.4 CI 95% 0.1–0.8).

The frequency of IDU was not included in the multiple GLMM to avoid overfitting. The conditional intraclass correlation (ICC) was 0.067, indicating no strong correlation between outcomes from individuals in the same prison.

The multiple GLMM showed that the highest odds ratios were associated with IDU (Table 1).

## Discussion

This was the first multicentre study in Belgian prisons screening prisoners for BBV using a finger prick test. Six percent of the screened prison population tested positive for HCV Ab, HBsAg, or HIV Ab/Ag. In this multicentre study, the HCV Ab (5.0%) and RNA positive prevalence (2.1%) were several times higher than the previously estimated prevalence in the general Belgian population, equal to, respectively, 1.0 and 0.3% [7]. There is a paucity of up-to-date data on interventions to improve the HCV care cascade in prisoners, as the systematic review by Kornfli et al. clearly describes [37]. Our results contribute to closing this gap of knowledge about BBV in prisons.

In our study, the observed prevalence for HCV Ab is lower than in other countries in Europe, Northern America, Australia, and the pooled prevalence of 15.5% in Western Europe, though similar to the prevalence in neighbouring countries as France, United Kingdom, Switzerland, and Denmark [5, 38–40]. As our study also shows, IDU and sharing of associated paraphernalia are the main risk factors for HCV. However, only one in five prisoners identified themselves as an injecting drug user in our study. This percentage is similar to the percentage of people who inject drugs (PWID) found in two Italian studies (23%) [39, 40]. However, the prevalence of HCV Ab in these studies was twice as high (10%). This could be explained by the fact that the viremic prevalence in the general population in Italy is also higher than in Belgium, resulting in a higher prevalence within the prison [41]. In addition, if we take into account the country of birth of the prisoners, the majority were born in a country with low to moderate HCV viremia. This may have contributed to the relatively low prevalence of HCV Ab in Belgian prisons compared to other Western European countries [5].

In a systematic review by the ECDC, the prevalence of HBsAg in prison ranged from 0.3 to 25.2% [8]. Our results are similar to France, Ireland, and Finland but low compared to those found elsewhere. Our data are similar to the prevalence found in the Belgian population presenting at an emergency department [25].

Our results on HIV prevalence (0.2%) also reflect the prevalence of the general Belgian population (0.3%) rather than the estimated prevalence (5.0%) in prisons in Western Europe [26, 27]. Moreover, these prisoners were already aware of their status and received antiretroviral therapy. Nevertheless, antiretroviral treatment can be interrupted by arrest and detention, depending on its availability [29]. In both cases, the antiretroviral treatment was ongoing. Therefore they were not further monitored in the study.

No coinfections were detected in this study. The prevalence of BBV coinfections in Western European prisons is relatively low, ranging from 0 to 0.4% in France, the United Kingdom, and Switzerland [42–45].

Prisoners with a positive HCV Ab or HBsAg rapid test were sent to a specialist for further follow-up after confirmation of infection via a regular blood sample. Five out of the seven prisoners with positive HBsAg were not aware of their status. Of the 19 prisoners with positive HCV RNA, nine (47.4%) were unaware of their infectious status. Moreover, this finding stresses the importance of screening, not only to identify new cases but also to identify previously known cases and treat them. Especially since the majority are often PWID, also in our study. This key population often exhibits persistent risk behaviour and thus causes further transmission [40].

For further follow-up on HCV and/or HBV, the prisoners were referred to one of the three Belgian prisons with an extensive medical service and specialists’ presence. However, we did not determine in retrospect how many prisoners started treatment. This lack of knowledge is a shortcoming of this study. Indeed, it has been shown in several studies that treating prisoners within prison walls is highly effective and feasible [46–48]. One study even concluded that incarceration does not affect unplanned interruptions or SVR rates in short-term therapies. Short schedules with pangenotypic regimens could be a valid approach for hard-to-reach populations, such as patients in captivity [49].

This study has several limitations. First, we used a convenience sample, which means that all prisoners in the pre-determined prisons (and sometimes pre-determined units) who were willing to participate were included in the study. Therefore, the risk of selection bias cannot be excluded. For example, prisoners who were aware of being infected or suspected of being infected because of previous or current IDU could have refused participation because they considered it unnecessary or because they were afraid of stigmatization. Second, we were unable to identify risk factors for HBV and/or HIV infection due to the low number of infections. Third, our study’s screening uptake ranged from 16.9 to 35.4% in long-term facilities. The relatively low uptake in long-term facilities can be due to the opt-in system used in this study. It is therefore strongly recommended to use an opt-out system [17]. Even though we were able to screen 886 prisoners, we did not achieve the predefined sample size for cluster design. However, the conditional ICC was 0.067, indicating no strong clustering within prisons and hence the obtained sample size can be deemed sufficient. In addition, in some prisons, sections were chosen in advance to participate in the study, which meant that not all prisoners could enrol. Furthermore, fear of stigmatization or lack of knowledge about the BBV may also have contributed to the prisoners’ decision not to participate. Finally, the data were derived using a face-to-face questionnaire that could have led to social desirability bias, which could have caused underreporting of high-risk behaviour.

## Conclusions

In conclusion, the HCV Ab prevalence in Belgian prisons is relatively low compared to prevalences worldwide though similar to surrounding countries. HBsAg and HIV prevalences in Belgian prisons are more similar to those found in the general Belgian population and lower than those reported in Western European prisons. To avoid selection bias and get an overview of the total infected prison population in a country, we urge the need for systematic screening of all prisoners via opt-out.

## Supplementary Information

**Additional file 1.**

## Data Availability

All data generated or analysed during this study are included in this published article.

## Abbreviations

HCV: Hepatitis C virus
WHO: World Health Organization
Ab: Antibody
DAA: Direct-acting antiviral
HBV: Hepatitis B virus
ECDC: European Center for Disease Prevention and Control
HBsAg: Hepatitis B surface antigen
BBV: Blood-borne viral infections
UH: Hasselt University
IDU: Injecting drug use
GLMM: Generalized linear mixed model
