# Supplementary figures and images for: A multicentre interventional study to assess blood-borne viral infections in Belgian prisons
Source: BMC Infect Dis. 2021 Jul 27;21:708. doi: 10.1186/s12879-021-06405-z (PMC8314587; doi:10.1186/s12879-021-06405-z)

**Additional file 1**


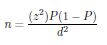
**A1. Formula for sample size calculation**

Supplement: Supplementary file 1 — Additional file 1. [file 12879_2021_6405_MOESM1_ESM.docx]
